# Types of Eating Disorder Prodrome in Adolescence: The Role of Decision Making in Childhood

**DOI:** 10.3389/fpsyg.2022.743947

**Published:** 2022-03-16

**Authors:** Amy Harrison, Marta Francesconi, Eirini Flouri

**Affiliations:** Department of Psychology and Human Development, Institute of Education, University College London, London, United Kingdom

**Keywords:** eating disorders, decision making, epidemiology, longitudinal, childhood, adolescence

## Abstract

Psychiatric disorders like eating disorders (EDs) might be underpinned by differences in decision making. However, little previous research has investigated this potential relationship using longitudinal data. This study aimed to understand how components of decision making (delay aversion, risk adjustment, risk taking, quality of decision making and deliberation time) measured by the Cambridge Gambling Task in the United Kingdom’s Millennium Cohort Study (MCS; *n* = 11,303; female = 50.17%) at age 11 might explain clusters/types of ED prodrome involving body dissatisfaction, intention to lose weight, dietary restraint, excessive exercise and significant under/overweight measured in the MCS at age 14. Latent class analysis revealed two groups within the cohort: a non-prodromal eating pathology group, who were more likely to be of “average” weight, according to the UK90, with minimal disordered attitudes and behaviors in relation to eating and weight; and a second group with prodromal eating pathology, who had more body dissatisfaction, a desire to lose weight, were using dietary restriction and exercise to influence weight and were more likely to be “overweight” according to the UK90. Logistic regression showed that, after adjustment for confounding, higher risk-taking scores were associated with a 60% greater probability of being in the prodromal eating pathology group (*b* = 0.47, OR = 1.60, *p* < 0.01), and higher scores on quality of decision making were associated with a 30% lower probability of being in the prodromal eating pathology group (*b* = −0.34, OR = 0.70, *p* < 0.05). Helping young people to engage in moderate risk taking and improving decision making might reduce the later presence of ED prodromes.

## Introduction

Eating disorders (EDs) such as anorexia nervosa (AN), bulimia nervosa (BN) and binge eating disorder (BED) are life-threatening psychiatric disorders which negatively impact psychological, physical and social functioning (Schmidt et al., 2016). A body of cross-sectional research [reviewed by Harrison et al. (2010)]—focused largely on adult, female, clinical samples – has found people with EDs make different decisions under conditions of risk compared to unaffected peers. Those with largely restricting symptoms, for example, as seen in AN, tend to be risk averse and cautious during decision-making, experience high anxiety and are driven to avoid aversive states, like exposing themselves to possible failure and making mistakes. Individuals who experience binging and/or purging behaviors, as seen in BED and BN, are also anxious and have high loss aversion, but also exhibit impulsivity and have high appetitive motivation (Harrison et al., 2010). These profiles can present challenges for this population and, as putative personality components (Gray and McNaughton, 2000), seem to change rather little even after intensive outpatient treatment (Harrison et al., 2016).

Our previous work using the Cambridge Gambling Task (CGT; Rogers et al., 1999; Deakin et al., 2004) has added to this literature (Harrison et al., in press) using data from the United Kingdom’s population-based Millennium Cohort Study (MCS). This work has involved using longitudinal data to explore how children and adolescents make decisions under conditions of risk using data from boys and girls in the MCS. We found that the ability to make advantageous decisions under conditions of risk at age 11 in childhood is associated with a lower probability of prodromal eating pathology in adolescence age 14. Specifically, those with better quality of decision making were 34% less likely to show an intention to lose weight (*b* = −0.40, OR = 0.66, *p* < 0.05) and 34% less likely to be “overweight” according to the UK90 (Cole et al., 1995; *b* = −0.41, RRR = 0.66, *p* < 0.05). Those who showed higher risk taking were 58% more likely to show dietary restriction (*b* = 0.45, OR = 1.58, *p* < 0.05) and 46% more likely to report excessive exercise (*b* = 0.38, OR = 1.46, *p* < 0.05). In the complete cases sample (where we considered only participants with full data, i.e., no missingness), higher risk adjustment (a tendency to stake higher bets on favorable compared to unfavorable trials) was associated with a 47% increased risk of being “underweight” according to the UK90 (Cole et al., 1995; *b* = 0.39, RRR = 1.47, *p* < 0.05), and better quality of decision making was associated with a 46% lower risk of being “overweight” according to the UK90 (Cole et al., 1995; *b* = −0.60, RRR = 0.54, *p* < 0.05). This suggests that individuals who, during childhood, show less advantageous decision-making skills, may be vulnerable to developing prodromal eating pathology in adolescence. A prodrome is an early symptom, or cluster of symptoms, which emerge before the onset of a syndrome, illness or disorder. Prodromes are distinct from risk factors because rather than indicating elevated risk for the presence of a disorder in future, a prodrome indicates the initiation of the onset of the disorder (Stice et al., 2010). Understanding more about prodromes is an approach that has been of use in schizophrenia (Larson et al., 2010), another long-term psychiatric disorder with high social disability similar to EDs (Wiersma et al., 2000; van Hoeken and Hoek, 2020). Indeed, our field may benefit from drawing on learning in schizophrenia, where being able to identify early signs of pathognomonic symptoms has enabled clinicians to intervene and prevent individuals from developing more insidious clinical syndromes (Correll et al., 2018).

In the current study, we were therefore interested in addressing new research questions in order to develop the evidence base relating to prodromal eating pathology. In this investigation, we aimed to examine how the pathognomonic prodromal ED symptoms of body dissatisfaction, intention to lose weight, dietary restriction, excessive exercise and significant “under” or “overweight,” according to the UK90 (Cole et al., 1995) might cluster together (for example, dietary restriction and low weight) within the sample with the aim of identifying possible types of ED prodrome at age 14. We further aimed to understand the relationship between decision making (delay aversion, risk adjustment, risk taking, quality of decision making and deliberation time) measured using the CGT at age 11 and types of ED prodrome at age 14.

This new analytic approach involving identifying possible types of ED prodrome adds to our previous work (Harrison et al., in press) because it allows us to understand the ways in which ED symptoms begin to cluster together in adolescence in this large community-based cohort. It also helps us to understand whether differences in decision making under conditions of risk might explain the presence or absence of specific ED prodromes. It is important to note that this work aims to move the focus away from clinical samples, such that we were not interested in identifying clinical diagnoses, but rather the real-world presence of symptoms which might point to the emergence of prodromal eating pathology in adolescence in a large community sample.

We aimed to address a research question around whether it would be possible to identify specific ED prodromes within the cohort. It was hypothesized that differences in decision-making (delay aversion, risk adjustment, risk taking, quality of decision making and deliberation time) measured using the CGT at age 11 would be associated with the presence versus absence of clusters of attitudes and behaviors reflecting prodromal eating pathology. Based on our previous work within the cohort (Harrison et al., in press), we expected, for example, that greater risk taking and lower quality of decision making would be associated with an ED prodrome.

## Materials and Methods

### Design

This prospective, observational, population-based cohort study uses a longitudinal design.

### Participants

The Millennium Cohort Study (MCS) is an ongoing cohort study developed as a multidisciplinary survey designed to explore the influence of early family context on child development and outcomes throughout childhood, into adolescence and subsequently through adulthood.^1^ To date, it has enrolled 19,244 families whose children were born across England, Scotland, Wales, and Northern Ireland in 2000–2002. The sample design over-represented families living in areas of high child poverty, areas with high proportions of ethnic minority populations across England and the three smaller United Kingdom countries. There have been data from six sweeps to date. MCS children were around 9 months old at Sweep 1, and 3, 5, 7, 11, and 14 years old at Sweeps 2–6, respectively. Data from sweeps 5 and 6 are used in this study: in the MCS, ED related attitudes and behaviors were measured at age 14 and CGT was measured at ages 11 and 14. The attrition rate between Sweeps 1 and 6 was 36.7%. Our analytic sample included singletons and first-born twins or triplets with available information on eating disorder related attitudes and behaviors at age 14 and with available CGT data at age 11 or 14 (*n* = 11,303). Ethical approval for sweeps 5 and 6 of the MCS was obtained from the Yorkshire and The Humber—Leeds East (ref. 11/YH/0203) and London Central (ref. 13/LO/1786) National Health Service Research Ethics Committees, respectively. The authors assert that all procedures contributing to this work comply with the ethical standards of the relevant national and institutional committees on human experimentation and with the Helsinki Declaration of 1975, as revised in 2008.

### Measures

#### Decision Making Under Conditions of Risk

The Cambridge Gambling Task (CGT) assesses decision-making and risk-taking behavior outside a learning context under conditions of uncertainty (Rogers et al., 1999; Deakin et al., 2004). The CGT has been widely used on children and adolescents in previous studies (Van Leijenhorst et al., 2008; Flouri et al., 2019). Participants view a computer monitor on which a total of 10 boxes (red and blue) appeared in varying ratios (6:4, 7:3, 8:2, 9:1) of red to blue. Participants track a yellow token hidden inside one of these boxes. They have to choose (a) which color of box they believe the token is hidden behind (red or blue), and (b) the number of points they want to gamble. The five CGT measures of decision making used in this study include: (1) the average number of points placed on bet after the most likely outcome was chosen (*risk-taking*); (2) the mean proportion of trials where the most likely color outcome was selected (*quality of decision-making*); (3) the mean reaction time for making a selection (*deliberation time*); (4) the tendency to stake higher bets on favorable compared to unfavorable trials (*risk adjustment*); and (5) the total difference between risk-taking scores (points gambled) in five different amounts of wagers (5, 25, 50, 75, and 95%) presented in either ascending or descending order (*delay aversion*). A sixth CGT measure, overall proportion bet, was excluded from our analysis in view of its very high correlation (>0.90) with the risk-taking variable. In the MCS, the Cronbach’s *α* of the CGT subscales is 0.93, reflecting an excellent reliability (Atkinson, 2015).

#### Prodromal Eating Pathology

Symptoms indicating the emergence of ED thoughts and behaviors were measured at age 14 using the available eating, dieting and body image questions from the Millennium Cohort Study. These dichotomous questions measured: *body dissatisfaction* (whether the participant reported a perception of their body as being too overweight); *intention to lose weight* (the presence of a strong desire to lose weight); *dietary restriction* (whether the participant had ever actively eaten less to influence their shape/weight) and *excessive exercise* (whether the participant had ever exercised in a driven way in order to influence weight and shape). These items are similar to those used to address disordered eating in other large adolescent studies (Figueiredo et al., 2019). We also included an objective measure of underweight and “overweight” based on the most widely used reference panel, the UK90 (Cole et al., 1995), which is sensitive to sex and age and developed for the British population. Cut-offs were based on the age of the cohort member at the time of interview. The underweight cut-off point was the second centile and the “overweight” cut-off point was the 85th centile, as suggested by the UK90. These items were entered in the Latent Class Analysis (LCA) that we ran to identify types of prodromal eating pathology.

#### Confounders

We included a number of covariates known to be associated with decision-making ability and eating pathology (Pike and Walsh, 1996; Bruine de Bruin et al., 2007; Griffiths et al., 2017): gender, ethnicity (according to the United Kingdom census groups of white, black, Indian, Pakistani/Bangladeshi, mixed, or other), family poverty (below the poverty line or not), IQ (verbal and non-verbal), derived in the MCS at age 5 from three subscales of the British Ability Scales (BAS; Elliott et al., 1996), pubertal status at age 11 (Baker et al., 2012; breast growth or menstruation or hair on body for females, and voice change or facial hair or hair on body for males) and internalizing and externalizing symptoms at age 11 (Evans et al., 2017; Lc and Zuluaga, 2021). These were assessed using the mother-rated Strengths and Difficulties Questionnaire (SDQ; Goodman, 1997). The SDQ is a valid and reliable tool for measuring such symptoms in children. It consists of 20 “difficulties” items related to behavior (in the past 6 months), with each item scored on a 3-point scale (0 = “not true,” 1 = “somewhat true,” and 2 = “certainly true”). Items can be summed to form four scales (emotional, conduct, hyperactivity, and peer problems) or two (internalizing symptoms, the sum of the scores on the emotional and peer problems items, and externalizing symptoms, the sum of the scores on the conduct and hyperactivity problems items), which we used for this analysis. We also took into account an objective measure of excessive exercise using accelerometer data. In the MCS, cohort members who participated in the age 14 sweep were asked to wear accelerometer devices for two specified full days: one during the week and the other at the weekend. The accelerometer data from the MCS at age 14 expresses their output in “Euclidean Norm Minus One” (ENMO), which is the calculation of the average magnitude of dynamic acceleration, i.e., the vector magnitude of acceleration corrected for gravity. It separates movement and gravity components in the acceleration signal. In our study, we considered the upper decile of the mean acceleration distribution for the weekday and the weekend day as moderate to vigorous physical activity. This was included as a covariate in our models because accelerometer data were only available for a subset of the sample (*n* = 4,711).

### Data Analysis

All analyses were performed in STATA 16.0. In all models, the MCS sampling stratum was controlled to account for the disproportionate stratification of the MCS sample. To identify potential clusters of ED symptoms in the cohort, we performed a LCA using the ED symptom items (body dissatisfaction, intention to lose weight, dietary restriction, excessive exercise, and weight status). We ran 1–3 class models and used three goodness of fit indices to determine which of these models fits best: (1) The Bayesian information criterion (BIC); (2) the Akaike information criterion (AIC), and (3) the entropy of each model. Lower BIC and AIC values indicate better fit to the data. Entropy ranges from 0 to 1, with higher values indicating that the latent classes are clearly distinguishable. Maximum Likelihood with Missing Values (MLMV) was used to deal with missing data. MLMV aims to retrieve as much information as possible from observations containing missing values. Finally, we fitted two different sets of logistic regression models in order to explore the association between CGT measures and symptom clusters. In our sample 49.67% of participants had at least one missing value and the remaining 50.33% of them had complete information in all the investigated variables. Missingness on the covariates ranged between 0.1% (ethnicity) and 28.7% (risk adjustment at age 11). To handle this, we used multiple imputation by chained equations (MICE; 20 imputed datasets).

## Results

Table 1 provides descriptive data on the Cambridge Gambling Task, eating pathology and demographic variables for the entire sample. Half of our sample (50.17%) were females and the majority (67.01% of females and 49.15% of males) had shown puberty signs by age 11. A bigger proportion of our sample also had an ethnic white background (81.92%) and was above the threshold of poverty (76.02%).

**Table 1:** Descriptive statistics (unweighted data) in the analytic sample (*N* = 11,303).

**Demographic variables**	***N***	**%**
Puberty (female)	6,362	67.01
Puberty (male)	4,686	49.15
Female sex	5,671	50.17
Below poverty line	2,711	23.98
**Ethnicity**		
White	9,250	81.92
Mixed	323	2.86
Indian	308	2.73
Pakistani or Bangladeshi	867	7.68
Black or Black British	363	3.21
Other ethnic group	180	1.60
**Cambridge gambling task**	***N***	***M*** **(SD)**
CGT Risk taking, age 11	10,301	0.52 (0.16)
CGT Deliberation time, age 11	10,302	3329.90 (1328.43)
CGT Risk adjustment, age 11	8,052	1.02 (0.82)
CGT Delay-aversion, age 11	9,201	0.33 (0.19)
CGT Quality of decision-making, age 11	10,302	0.80 (0.16)
CGT Overall proportion bet, age 11	10,301	0.48 (0.15)
IQ, age 5	10,505	101.15 (14.81)
**Prodromal eating pathology**	***N***	**%**
Body dissatisfaction (Perception of weight as very overweight)	510	4.61
Intention to lose weight (A strong desire to lose weight)	4,659	42.05
Dietary restriction (Actively reducing nutritional intake to influence shape/weight)	4,935	44.62
Excessive exercise (Driven use of exercise to influence body weight/shape)	6,687	60.36
Underweight cut-off for child’s age and sex-significant underweight^*^	169	1.56
Overweight cut-off for child’s age and sex-significant overweight^*^	3,751	34.52

*N* *= number of participants with complete information on the listed variables in our analytic sample; CGT = Cambridge gambling task; *M* = mean; SD = standard deviation. Puberty = those who had shown signs of puberty at age 11*.

* *Based on the UK 90 (Cole et al., 1995)*.

### Latent Class Analysis

Table 2 shows the fit indices of the competing LCA models. Starting with the one-class model, manually stepwise forward additions of classes resulted in lower BIC and AIC values, suggesting better model fit to the data for higher-class solutions. However, the three-class model showed a lower entropy value compared to the two-class solutions. Therefore, we considered the two-class solution optimal.

**Table 2:** Goodness-of-fit statistics of the competing latent class analysis models.

	One class solution	Two class solution	Three class solution
Entropy		0.7831010	0.6317767
AIC	64912.40	54762.268	54312.289
BIC	64956.39	54857.595	54458.945

The latent class marginal means, which represent the probability of responding positively to each question investigating ED symptoms, are shown in Table 3. We found that it was more likely that those belonging to class 1: (a) did not have a perception of their weight as very overweight; (b) were not intending to try to lose weight; (c) were not actively restricting their nutritional intake to lose weight; (d) were not exercising to influence their body weight; and (e) were more likely to have a weight that was not in the UK90 “under” or “overweight” ranges. We defined this as the ‘non-prodromal eating pathology group.’

**Table 3:** Latent class marginal means.

Variable name	Margin	SD	95% CI
**Class 1—Non-prodromal eating pathology group**			
Body dissatisfaction	0.00	0.00	0.00–0.00
Dieting behaviors	0.04	0.00	0.03–0.05
Dietary restraint	0.08	0.00	0.07–0.09
Excessive exercise	0.28	0.00	0.26–0.29
**Significant under/Overweight cut-off**			
0 (Average)	0.84	0.00	0.82–0.85
1 (Underweight)	0.02	0.00	0.02–0.03
2 (Overweight)	0.12	0.00	0.11–0.14
**Class 2—Prodromal eating pathology group**			
Body dissatisfaction	0.08	0.00	0.08–0.09
Dieting behaviors	0.79	0.00	0.78–0.81
Dietary restraint	0.80	0.00	0.79–0.81
Excessive exercise	0.92	0.00	0.91–0.93
**Significant under/Overweight cut-off**			
0 (Average)	0.43	0.00	0.41–0.44
1 (Underweight)	0.00	0.00	0.00–0.00
2 (Overweight)	0.56	0.00	0.55–0.58

*SD = standard deviation. Weight cut-offs are taken from the UK90 (Cole et al., 1995)*.

On the contrary, it was more likely that those belonging to class 2: (a) had a perception of their weight as too overweight; (b) were intending to try to lose weight; (c) were restricting their nutritional intake to lose weight; (d) were exercising to influence their body weight; and (e) had an average, or above average (“overweight”) weight, according to the UK90. We defined this as the ‘prodromal eating pathology group.’

Table 4 shows the descriptive statistics of the study variables in the analytic sample and the differences between the mutually exclusive prodromal eating pathology and non-prodromal eating pathology groups identified using LCA.

**Table 4:** Distribution between classes of the study variables in the analytic sample (*n* = 11,303; unweighted).

	**Class 1—Non-prodromal eating pathology group (*n* = 5,640)**		**Class 2—Prodromal eating pathology group (*n* = 5,663)**		**Test**
	***N***	***M* (SD)**	***N***	***M* (SD)**	***F***
**Continuous variables**
Risk taking, age 11	5,157	0.53 (0.16)	5,144	0.52 (0.16)	8.31^**^
Quality of decision-making, age 11	5,158	0.81 (0.16)	5,144	0.79 (0.17)	17.62^**^
Deliberation time, age 11	5,158	3297.08 (1358.39)	5,144	3362.80 (1296.98)	6.31^*^
Risk adjustment, age 11	4,090	1.05 (0.82)	3,962	1.00 (0.81)	7.20^**^
Delay-aversion, age 11	4,609	0.33 (0.19)	4,592	0.33 (0.20)	1.78
IQ, age 5	5,250	101.3 (14.91)	5,255	100.98 (14.72)	1.35
Internalizing symptoms, age 11	5,234	2.98 (3.00)	5,211	3.36 (3.22)	39.20^**^
Externalizing symptoms, age 11	5,236	4.30 (3.51)	5,203	4.42 (3.51)	2.94
**Categorical variables**
	***N***	**%**	***N***	**%**	***χ*** ^**2**^
**Puberty signs (female)**					
Yes	2,799	58.8	3,563	75.16	284.98^**^
No	1,955	41.2	1,177	24.84	
**Puberty signs (male)**					
Yes	2,047	42.65	2,639	55.72	162.85^**^
No	2,752	57.35	2,097	44.28	
Gender-female	2,239	39.69	3,432	60.60	493.98^**^
Gender-male	3,401	60.31	2,231	39.40	
Below poverty line	1,275	22.60	1,436	25.35	11.73^**^
Above poverty line	4,365	77.40	4,227	74.65	
**Ethnicity**					
White	4,671	82.95	4,579	80.90	8.01^**^
Mixed	155	2.75	168	2.97	0.47
Indian	145	2.57	163	2.87	0.98
Pakistani and Bangladeshi	403	7.16	464	8.20	4.31^*^
Black or Black British	171	3.04	192	3.39	1.14
Other ethnic group	86	1.53	94	1.67	0.32
Upper decile physical activity (derived from accelerometers)	428	17.88	305	13.15	20.03^**^
Lower deciles physical activity (derived from accelerometers)	1,965	82.12	2,013	86.85	

* *p* *< 0.05;*

** *p < 0.01*.

The prodromal eating pathology group showed lower scores on risk taking, quality of decision making and risk adjustment at age 11. They also showed higher scores on deliberation time and had higher levels of internalizing symptoms. Moreover, they were more likely to fall below the poverty line. Finally, a higher proportion of white children and a lower proportion of Pakistani and Bangladeshi children comprised the non-prodromal eating pathology group.

### Logistic Regression Modeling

Table 5 shows the results of our logistic regression models. In model 1, the unadjusted model, we found that higher risk-taking scores were associated with a 31% lower probability of being in the higher risk group (*b* = −0.36, OR = 0.69, *p* < 0.05). In the fully adjusted model, we found that higher risk-taking scores were associated with a 60% higher probability of being in the prodromal eating pathology group (*b* = 0.47, OR = 1.60, *p* < 0.01) and that higher scores in quality of decision making were associated with a 30% lower risk of being in the prodromal eating pathology group (*b* = −0.34, OR = 0.70, *p* < 0.05).

**Table 5:** Predictive models of prodromal eating pathology.

	**Imputed cases (*n* = 11,303)**			
	***b***	**SE**	**95% CI**	**OR**
**Model 1**
Risk taking, age 11	−0.36^*^	0.16	−0.69 to −0.03	0.69
Quality of decision-making, age 11	−0.19	0.15	−0.49 to 0.11	0.82
Deliberation time, age 11	0.00	0.00	−0.00 to 0.00	1.00
Risk adjustment, age 11	−0.07	0.03	−0.14 to 0.00	0.93
Delay-aversion, age 11	0.18	0.13	0.08 to 0.45	1.20
**Model 2**
Risk taking, age 11	0.47^**^	0.18	0.10 to 0.84	1.60
Quality of decision-making, age 11	−0.34^*^	0.16	−0.66 to −0.02	0.70
Deliberation time, age 11	0.00	0.00	−0.00 to 0.00	1.00
Risk adjustment, age 11	0.01	0.03	−0.05 to 0.08	1.01
Delay-aversion, age 11	0.21	0.14	−0.07 to 0.50	1.24

*Model 1 = Cambridge Gambling Task measures. Model 2 = Model 1+ gender, ethnicity, family poverty status, IQ at age 5, puberty signs at age 11, exact age, accelerometer-measured physical activity at age 14, internalizing and externalizing symptoms at age 11. The table displays results of our logistic regression analyses*.

* *p* *< 0.05;*

** *p < 0.01*.

## Discussion

This study aimed to build on our previous findings which have shown how in the MCS, less advantageous responding on the CGT at age 11 was associated with the presence of prodromal eating pathology at age 14 (Harrison et al., in press). We sought to understand more about the nature of the prodromal eating pathology emerging in the cohort by age 14 with regard to how putative symptoms might cluster together to form types of prodrome. We then examined how decision making (delay aversion, risk adjustment, risk taking, quality of decision making, and deliberation time) measured by the CGT in the MCS at age 11 might explain the presence of ED prodromes [body dissatisfaction, intention to lose weight, dietary restraint, excessive exercise, and significant “under/overweight,” according to the UK90 (Cole et al., 1995)] measured in the MCS at age 14.

The latent class analysis identified two groups within the cohort: one without prodromal eating pathology, with those belonging to this group being more likely to be of “average” weight, according to the UK90, not report the presence of disordered attitudes and behaviors in relation to eating and weight; the other group showed evidence of prodromal eating pathology by the age of 14 and they had more body dissatisfaction, were intending to lose weight, were using dietary restriction and exercise to influence weight and were more likely to be “overweight,” according to the UK90 cutoffs than their non-prodromal peer group.

While the two-class solution was the answer to our research question relating to the identification of specific ED prodromes, we had thought we might identify more than one type of ED prodrome within the cohort. While other solutions which differentiated clusters of ED-related symptoms on the basis of theory (e.g., AN, BN, and BED) were considered, they had to be discarded in favor of the two class solution as they did not meet acceptable statistical criteria. Another explanation for there only being two clusters relates to the questions asked in the MCS, over which we had no control. Had there been a wider range of questions around eating pathology, we may have obtained different findings. This relates to an important implication of this work, which is that to be able to understand more about the development of ED prodromes in adolescence, and the factors that influence their presence, it is crucial for researchers in the ED field to influence the questions asked in future longitudinal studies. Another perspective, in support of the transdiagnostic model of EDs (Fairburn, 2008), is that during early adolescence a broad range of ED symptoms develops. In time, ED symptoms become more differentiated into specific clusters of later ED cognitions and habitual behaviors which start to look more like AN, BN, and BED, described in the psychiatric nomenclature.

It was hypothesized that differences in the CGT variables at age 11 would be associated with the presence versus absence of prodromal eating pathology at age 14. The fully adjusted models showed that higher risk-taking scores were associated with a 60% higher probability of being in the prodromal eating pathology group and higher scores in quality of decision making were associated with a 30% lower probability of being in the prodromal eating pathology group. This highlights the role played by higher risk-taking and poorer decision-making in prodromal eating pathology. It might be that one way of protecting against disordered eating would be to offer training in childhood to support moderate risk-taking and help young people to develop more advantageous decision-making skills. This is something we aim to explore in future research.

These findings provide further evidence for the importance of advances by computational psychiatry suggesting that atypical decision making may underlie many forms of psychopathology (Montague et al., 2012). They also add to the wider evidence around associations between decision-making in childhood and adolescent psychopathology. For example, a recent study, also using MCS, showed that risk-taking measured in childhood at age 11 was associated with depressive symptoms in adolescence at age 14 among females (Lewis et al., 2021).

The multi-purpose nature of the MCS means that we did not have a clinical interview for EDs available, an important limitation. However, the items are similar to those asked in screening tools like the SCOFF (Morgan et al., 1999). It is also important to note that clinical diagnosis was not the focus of this work and that, as discussed earlier, this study aimed to explore the emergence of symptoms which might indicate the beginnings of an ED in a large community cohort sample to improve the inclusion of a broader range of individuals in ED research. As discussed, we did not have influence over the types of questions asked in the MCS, nor the timing of them, which meant, for example, that we were unable to discern ED symptoms which might have been present at age 11 and control for these in the analysis. Ideally, we would have included the accelerometer data as one of the variables we examined for the clusters of ED symptoms rather than as a covariate. However, as these data were only available for a subgroup of participants at age 14 only who opted to wear the accelerometers, we decided to conservatively include it as a confounder, especially as the other measure of exercise used was a self-report. Another potential issue with the accelerometer data is that participants wore these purposively and therefore may have altered their behavior as they were aware their activity levels were being measured. Another limitation we must acknowledge is that while we used the recommended cut-offs for “underweight” and “overweight” from the UK90, the second centile likely underestimates the presence of underweight and is not directly equivalent to the 85th centile for “overweight” and this could explain why we find a relatively small number of individuals in the cohort who were significantly “underweight.” Finally, it was interesting to observe that the direction of effect of risk-taking changed from age 11 to age 14. This phenomenon is known as the Lord’s Paradox (Tu et al., 2008) and is relevant to situations where some of the covariates lie on a causal pathway. We found that adjusting for gender explained this effect.

## Conclusion

In conclusion, this study identified a prodromal eating pathology group within the MCS sample who are differentiated from their peers without prodromal eating pathology by the presence of body dissatisfaction, the intention to reduce their weight, their active dietary restriction, the use of exercise to influence their weight, and by being more likely to be “overweight,” according to the UK90 (Cole et al., 1995). Higher risk-taking and poorer quality of decision-making measured by the CGT at age 11 are significant predictors of belonging to the prodromal eating pathology group at age 14.

## Data Availability Statement

The dataset analyzed for this study can be accessed freely by other researchers through the Centre for Longitudinal Studies. Available at: https://cls.ucl.ac.uk/cls-studies/millennium-cohort-study/.

## Ethics Statement

Ethical approval for sweeps 5 and 6 of the Millennium Cohort Study was obtained from the Yorkshire and The Humber—Leeds East (ref. 11/YH/0203) and London Central (ref. 13/LO/1786) National Health Service Research Ethics Committees, respectively. Written informed consent to participate in this study was provided by the participants’ legal guardian/next of kin.

## Author Contributions

AH and EF designed the study. MF analyzed the data. AH drafted the manuscript. MF and EF contributed to the final manuscript. All authors contributed to the article and approved the submitted version.

## Funding

This work was supported by a Medical Research Council grant (ref. MR/S019707/1) awarded to AH, Principal Investigator and EF, Co-Investigator.

## Conflict of Interest

The authors declare that the research was conducted in the absence of any commercial or financial relationships that could be construed as a potential conflict of interest.

## Publisher’s Note

All claims expressed in this article are solely those of the authors and do not necessarily represent those of their affiliated organizations, or those of the publisher, the editors and the reviewers. Any product that may be evaluated in this article, or claim that may be made by its manufacturer, is not guaranteed or endorsed by the publisher.

## Footnotes

^1^ www.cls.ioe.ac.uk/mcs
